# Cryo-EM structure and function of *S. pombe* complex IV with bound respiratory supercomplex factor

**DOI:** 10.1038/s42004-023-00827-3

**Published:** 2023-02-16

**Authors:** Agnes Moe, Pia Ädelroth, Peter Brzezinski, Linda Näsvik Öjemyr

**Affiliations:** 1 Department of Biochemistry and Biophysics, The Arrhenius Laboratories for Natural Sciences, Stockholm University, SE-106 91 Stockholm, Sweden; 2 Present Address: Xbrane Biopharma AB, Retzius väg 8, SE-171 65 Solna, Sweden

**Keywords:** Biophysical chemistry, Cryoelectron microscopy, Oxidoreductases

## Abstract

Fission yeast *Schizosaccharomyces pombe* serves as model organism for studying higher eukaryotes. We combined the use of cryo-EM and spectroscopy to investigate the structure and function of affinity purified respiratory complex IV (CIV) from *S. pombe*. The reaction sequence of the reduced enzyme with O_2_ proceeds over a time scale of µs-ms, similar to that of the mammalian CIV. The cryo-EM structure of CIV revealed eleven subunits as well as a bound hypoxia-induced gene 1 (Hig1) domain of respiratory supercomplex factor 2 (Rcf2). These results suggest that binding of Rcf2 does not require the presence of a CIII-CIV supercomplex, *i.e*. Rcf2 is a component of CIV. An AlphaFold-Multimer model suggests that the Hig1 domains of both Rcf1 and Rcf2 bind at the same site of CIV suggesting that their binding is mutually exclusive. Furthermore, the differential functional effect of Rcf1 or Rcf2 is presumably caused by interactions of CIV with their different non-Hig1 domain parts.

## Abstract (web summary)

Fission yeast *Schizosaccharomyces pombe* shares many characteristics with higher eukaryotes. Here, the authors investigate the structure and function of respiratory complex IV from *S. pombe*, reveal the subunit arrangements and the reaction sequence of O_2_ reduction.

## Introduction

In the final steps of cellular energy conversion electrons are transferred from NADH to O_2_, through a series of membrane-bound protein complexes, referred to as the respiratory chain. The energy released during this process is utilized to translocate or pump protons across the membrane thereby maintaining a proton-electrochemical gradient that drives ATP synthesis as well as other transport processes in the cell. In aerobic bacteria the respiratory complexes are found in the cytoplasmic membrane while in eukaryotes they are located in the inner mitochondrial membrane (for review, see^1^).

The final O_2_-reducing complex of the respiratory chain, cytochrome *c* oxidase, also known as complex IV (CIV), receives electrons from soluble cytochrome *c* (cyt. *c*), which is reduced by the cyt. *bc*_1_ complex, CIII. In CIV electrons are first transferred to the primary electron acceptor, Cu_A_, and then consecutively to heme *a*, and the heme *a*_3_-Cu_B_ catalytic site where oxygen is reduced to water. Protons for water formation are transferred from the matrix of mitochondria, i.e. the negative (*n*) side of the membrane, through proton pathways that span the distance between the *n*-side solution and the catalytic site. In addition, each electron transfer to the catalytic site is linked to pumping of one proton across the membrane, from the *n* to the positive (*p*) side, i.e. the intermembrane space in mitochondria (for review, see^2^).

The first high resolution X-ray crystal structures of mammalian^3^ and bacterial^4^ CIV were published over 20 years ago. They show a conserved catalytic core composed of subunits I-III (Cox1-3 in yeast), including conserved lipid sites^5,6^. Subunit I harbors redox cofactors heme *a*, heme *a*_3_ and Cu_B_, subunit II binds the primary electron acceptor, Cu_A_. In addition, the mitochondrial CIV is composed of several supernumerary subunits. The *Saccharomyces* (*S*.) *cerevisiae* CIV, which serves as a model for studies of the mammalian CIV^7^, is composed of nine supernumerary subunits, eight identified originally^8^ and the yeast-specific Cox26, identified more recently^9–12^. Cryo-EM structures of the *S. cerevisiae* CIV, as part of a supercomplex with CIII_2_ (CIII_2_CIV_1/2_) have been presented^11–15^ and a structure of isolated CIV, without canonical subunits, Cox12 and Cox13, as well as Cox26, has been described^16^.

Fission yeast *Schizosaccharomyces* (*S*.) *pombe* serves as an alternative model organism for studies of the mammalian respiratory chain^17^. Procedures for genetic manipulations have been established and a wide range of molecular tools are available for studies of this fungus^18^. *S. pombe* and *S. cerevisiae* are evolutionary highly diverged from each other and neither of them is closer to mammals than the other^17,19,20^. Nonetheless, *S. pombe* retains many features of the common ancient yeast ancestor and *S. pombe* therefore shares more characteristics with higher eukaryotes than *S. cerevisiae*^17,21–24^. As a result, this fungus has been described as “the model unicellular eukaryote”^21^. Yet, to our knowledge, structural or mechanistic studies of components of the respiratory chain at a molecular level have not previously been performed with *S. pombe*.

The *S. pombe* CIV is composed of 11 subunits, which share many sequence similarities with the *S. cerevisiae* CIV counterparts (Table 1). Just like *S. cerevisiae*, *S. pombe* lacks respiratory CI, and the genes of the membrane-bound core subunits of CIII (*cob*), CIV (*cox1-3*) and ATP synthase (*atp6,8-9*) are located on the mitochondrial DNA^22^. In addition to the three core subunits, the *S. pombe* CIV has annotated homologs for eight of the supernumerary nuclear-encoded subunits (PomBase, pombase.org), except Cox26. The *S. pombe* CIV contains only one gene for subunit Cox5, compared to the two isoforms Cox5a/b in *S. cerevisiae*^7,25^.

**Table 1:** Annotated *S. pombe* CIV subunits.

Subunit			
*S. pombe*	*S. cerevisiae*	Identity (%)	Similarity (%)
COX1_SCHPO	COX1_YEAST	61 (325/535)^a^	
COX2_SCHPO	COX2_YEAST	54 (130/241)^a^	
COX3_SCHPO	COX3_YEAST	48 (126/265)^a^	
COX4_SCHPO	COX4_YEAST	50^b^	66
COX5_SCHPO	COX5A_YEAST	34^b^	50
	COX5B_YEAST	44^b^	58
COX6_SCHPO	COX6_YEAST	53^b^	71
COX7_SCHPO	COX7_YEAST	38^b^	59
COX8_SCHPO	COX8_YEAST	33^b^	47
COX9_SCHPO	COX9_YEAST	41^b^	59
COX12_SCHPO	COX12_YEAST	55^b^	70
COX13_SCHPO	COX13_YEAST	38^b^	58

The *S. pombe* subunits from pombase.org (GO:0005751) are compared with corresponding subunits of the *S. cerevisiae* CIV. Degree of amino acids sequence identity for the ^a^mitochondrial encoded subunits from BLAST search at https://blast.ncbi.nlm.nih.goc/Blast.cgi and ^b^amino acid sequence identity and similarity for nuclear encoded subunits is from https://flyrnai.org.dipot-dist.

The so-called respiratory supercomplex factors (Rcfs)^26–28^ are integral membrane proteins, located in the inner mitochondrial membrane. These proteins are members of the conserved hypoxia-induced gene 1 (Hig1) protein family^26^ and have been suggested to be involved in the assembly of the CIII_2_CIV_1/2_ supercomplex as well as that of CIV^28–31^. The yeast-specific Rcf2 in *S. cerevisiae* is a 25 kDa protein that was predicted to be composed of four transmembrane (TM) α-helices^29,32^ and it was shown to form a dimer in DPC micelles^33^. A recent cryo-EM study of the *S. cerevisiae* CIII_2_CIV_1/2_ supercomplex with the two Cox5a or 5b isoforms, identified Rcf2 in CIV harboring the hypoxia-induced Cox5b isoform supercomplex, bound at the distal part of CIV with two TM α-helices interacting with subunits Cox3 and Cox13, and one additional helix bound to Cox12 on the *p* side of CIV^13^.

In this work we added a C-terminus twin-strep tag on subunit Cox5 and affinity purified the *S. pombe* CIV. We investigated ligand binding to the enzyme as well as the reaction of the reduced CIV with O_2_. The data show the same general reaction sequence as that observed previously with other canonical CIV. Determination of the cryo-EM structure of CIV identified eleven subunits as well as Rcf2 in the same position as that found in the recent structure of the *S. cerevisiae* CIV supercomplex^13^, which shows that Rcf2 is a component of CIV regardless of the Cox5a/5b isoforms and not only in the III-IV supercomplex.

## Results and Discussion

### Isolation and activity

We constructed a C-terminus twin-strep tag on subunit Cox5 of *S. pombe* CIV and solubilized the membrane fragments using the detergent glyco-diosgenin (GDN) followed by affinity chromatography. Two protein populations were separated on a size exclusion column (supplementary Fig. S1a). Analysis of the reduced minus oxidized difference spectra of these populations showed that the lower molecular weight population (peak 2, red – supplementary Fig. S1b) had absorbance maxima at 445 nm and 607 nm characteristic of CIV. The higher molecular weight population (peak 1, black) contained also CIII (absorbance maxima at 430 nm, 550 nm and 560 nm) in addition to CIV, at CIII:CIV ratio of ∼2:1, in agreement with a CIII_2_CIV_1_ supercomplex.

The activity of CIV was determined by measuring the O_2_-reduction rate upon addition of cyt. *c*, which was maintained in a reduced state by an excess of ascorbate and the electron mediator N,N,N’,N’-tetramethyl-*p*-phenylenediamine (TMPD). At pH 6.5 this activity was 340 ± 80 e^–^ s^–^^1^ (eight measurements, three independent preparations), i.e. in the same range as that of *S. cerevisiae* CIV^34^.

The GDN-purified CIV yielded a homogenous preparation suitable for structural studies using cryo-EM and steady state oxygen reduction assays. However, in GDN only a fraction of CIV was extracted and, as indicated above, it partly co-purifies in a supercomplex with CIII. Consequently, the yield of pure CIV was not sufficient to perform functional analyses using time-resolved optical absorption spectroscopy. Therefore, we instead used the detergent n-dodecyl-β-D-maltoside (DDM), commonly used in purification strategies of CIVs from other species, for solubilization and purification for these experiments. Purification of CIV using DDM rendered a preparation free from CIII (supplementary Fig. S1de) and a yield that was sufficient for functional studies. The activity in DDM was slightly lower than that in GDN, 200 ± 40 e^−^ s^−^^1^ (*n* = 8, three independent preparations), which could be caused by missing accessory subunits Cox12 and Cox13 (see below).

### Structure determination by Cryo-EM

**Fig. 1:**
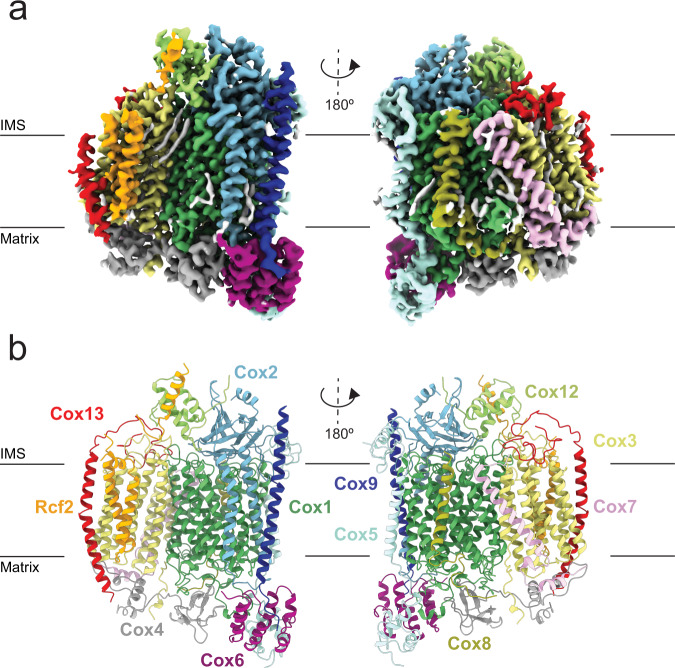
Cryo-EM structure of the *S. pombe* CIV. The approximate position of the membrane is indicated, IMS denotes intermembrane space. **a** Cryo-EM map. **b** Atomic model.

Cryo-EM of the *S. pombe* CIV purified in GDN yielded a map with an overall resolution of 3.4 Å (Fig. 1, supplementary Fig. S2 and Table 2). The map allowed construction of an atomic model (supplementary Fig. S3) containing Cox1-9, Cox12 and Cox13, with overall structural similarity to CIV of the *S. cerevisiae* CIII-CIV supercomplexes^11,13,14^. We identified an additional density attributed to the two TM α-helices of Rcf2 (see below). BN-PAGE bands subjected to mass spectrometry identified Rcf2 in CIV purified in GDN, but not in CIV purified in DDM (supplementary Fig. S1).

**Table 2:** Cryo-EM data collection, refinement and validation statistics.

**Data collection and processing**	
Magnification	105,000
Voltage (kV)	300
Electron exposure (e^−^/Å^2^)	49
Defocus range (μm)	0.6–2.6
Pixel size (Å)	0.86
Symmetry imposed	None (C1)
Initial particle images (no.)	2,609,959
Final particle images (no.)	127,659
Map resolution (Å)	3.36
FSC threshold	0.143
Map resolution range (Å)	1.6–6.5
**Refinement**	
Model resolution (Å)	3.6
FSC threshold	0.5
Map-sharpening *B* factor (Å^2^)	126.1
Model composition	
Nonhydrogen atoms	14,787
Protein residues	1824
Ligands	11
B factors (Å^2^)	
Protein	71.43
Ligand	60.75
Root mean square deviations	
Bond lengths (Å)	0.003
Bond angles (°)	0.807
Validation	
MolProbity score	1.6
Clashscore	4.51
Poor rotamers (%)	0.2
Ramachandran plot	
Favored (%)	94.49
Allowed (%)	5.45
Disallowed (%)	0.06

As seen in supplementary Fig. S4 the two isoforms Cox5a/b in *S. cerevisiae*^7,25^ and the single isoform Cox5 in *S. pombe* have similar structures and a high degree of sequence homology, except at the C- and N-termini where the *S. pombe* Cox5 carries additional residues not present in the *S. cerevisiae* Cox5a/b.

The cryo-EM analysis revealed a minor subpopulation of CIV missing Cox12, Cox13 and Rcf2, which is similar to the *S. cerevisiae* structural model of CIV isolated in the detergent n-undecyl-β-D-maltoside (UDM), then exchanged for amphipols^16^. This observation indicates that subunits Cox12 and Cox13 as well as Rcf2 are loosely bound and may be lost during purification of CIV, which is supported by previous data^26,28,35^.

**Fig. 2:**
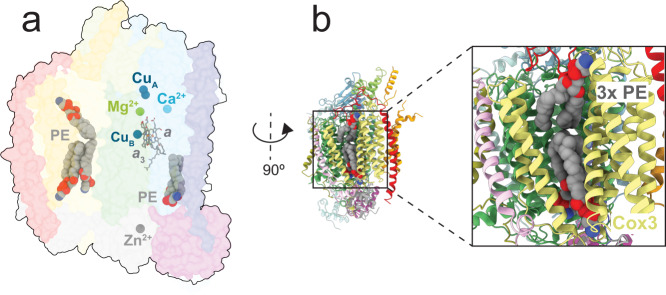
Lipids, redox-active cofactors and non-redox-active metal sites. **a** Density for four phosphatidylethanolamine (PE) molecules was clearly resolved. The redox-active metal sites are Cu_A_, heme *a* as well as the heme *a*_3_-Cu_B_ catalytic site. Non-redox-active Zn^2+^, Ca^2+^ and Mg^2+^ sites were modelled as discussed in the text. **b** Three PE lipids in the V-shaped cleft of Cox3 (see text).

All redox-active metal cofactors were found at the same position as in other canonical CIVs (Fig. 2a). As discussed above, the electron acceptor from cyt. *c* is a di-nuclear Cu_A_ site in Cox2. From Cu_A_ electrons are transferred consecutively to heme *a* and the heme *a*_3_-Cu_B_ catalytic site found in subunit Cox1. As with other canonical CIVs, we identified a covalent link between His247 and Tyr251 at the catalytic site. Conserved protonatable residues involved in proton transfer, via the D and K proton pathways displayed similar positions as in structures of other canonical CIV^1,7^.

In addition to the redox-active cofactors, density was observed for several non-redox active protein-bound metal ions (Fig. 2a), consistent with earlier observations with mammalian and bacterial CIV. An Mn^2+^/Mg^2+^ site (depending on the metal content of the growth medium), modelled here as Mg^2+^, is identified near the catalytic site^3,11,36,37^. A Ca^2+^/Na^+^ site, modelled here as Ca^2+^, is found in subunit Cox1^3,11,37,38^. In addition, a Zn^2+^ ion is modelled in Cox4^3,11^.

As with the *S. cerevisiae* and many other CIV, in *S. pombe* subunit Cox 3 is composed of seven TM α-helices that form a V-shaped cleft defined by a two- and a five-helix bundle, respectively. The cleft harbors tightly bound lipid molecules, resolved in crystal and cryo-EM structures of CIV from e.g. *B. taurus*, *P. denitrificans*, *R. sphaeroides* or *S. cerevisiae*^6,11,13^. In the current *S. pombe* structural model three lipids were modelled in Cox3 as phosphatidylethanolamine (PE) based on map interpretation and similarities with previous structures (Fig. 2b). Density for one additional PE molecule was found in between the two transmembrane helices of Cox2, in a similar position as previously found in *S. cerevisiae* CIV^11,13^. In the studies of the *S. cerevisiae* supercomplex, several lipids were also found at Cox1 and Cox5, near or at the interface with CIII. In the *S. pombe* CIV we identified diffuse lipid densities at a similar position, i.e. at the surface of Cox1 and Cox5. However, these densities did not occupy the same positions as in the *S. cerevisiae* supercomplex and they were too weak to be satisfactorily modelled.

Cryo-EM structures of *S. cerevisiae* supercomplexes identified also the Cox26 subunit^11,12^. However, in *S. pombe* there is no homologous protein annotated and there is no density in the corresponding position in our CIV structure. Furthermore, a protein previously assigned as a complex I subunit, NDUFA4, was suggested to be a CIV subunit^39,40^ (but see ref. ^41^). In *S. pombe* gene SPCC417.16 is annotated as an orthologue to the Ndufa4A protein, however, no density was observed that could account for this protein.

### Binding of the respiratory supercomplex factors

Three Rcf variants, Rcf1-3 have been identified and originally suggested to be involved in formation of the CIII-CIV supercomplex in *S. cerevisiae*^26–29,42–44^. However, more recent data instead suggest that Rcf1 is involved in assembly and regulation of CIV^30,45–47^.

Rcf1 and Rcf2 contain a conserved hypoxia-induced gene domain 1 (HIG1)^26,48–51^ (supplementary Fig. S5). Rcf2 is a fungi-specific protein in which the HIG1 domain is located at the C-terminus. In addition, Rcf2 harbors a sub-domain composed of ~100 amino-acid residues, which form two TM α-helices at the N-terminus^32,33^. The recently determined cryo-EM structure of the *S. cerevisiae* Cox5b-containing supercomplex identified the two HIG1-domain TM α-helices of Rcf2 bound mainly to Cox3, but also interacting with Cox13^13^, consistent with results from biochemical studies^26,28,30,52,53^. An additional C-terminal part of Rcf2 formed an α-helix found bound to Cox12 at the *p*-side of the membrane (intermembrane space).

We identified a density attributed to two TM α-helices in the *S. pombe* CIV at the same position as in the *S. cerevisiae* CIII_2_CIV_1/2_ supercomplex. This density could be convincingly modelled as the two HIG1 TM α-helices of Rcf2. Density for an α-helix near Cox12 at the *p*-side of the membrane that we attribute to Rcf2 is also seen in *S. pombe* CIV. However, it lacks sufficient resolution to identify the amino acid side chains and was therefore modelled as a polyalanine peptide. The connection between this helix and the transmembrane part of Rcf2 was not resolved. The *S. pombe* Rcf2 contains an additional 21 residues loop (supplementary Fig. S5), not present in *S. cerevisiae*, between the transmembrane HIG1 domain and the α helix near Cox12. This additional loop is not seen in the cryo-EM map and therefore presumably flexible. As with the structure of the *S. cerevisiae* supercomplex with bound Rcf2, the *S. pombe* N-terminal domain of Rcf2 was not resolved. This observation suggests that it is the processed, C-terminal form of Rcf2^13,29^ that forms part of the mature CIV. Furthermore, the data suggest that the C-terminal HIG1 domain of Rcf2 does not only associate with the CIII-CIV supercomplex, but also with monomeric CIV (see also^13,29,51^).

The Hig1 family can be divided into two subgroups, types 1 and 2, where both Rcf1 and Rcf2 are part of the Hig1 type 2 subgroup. This subgroup is characterized by the presence of a so called QRRQ motif (supplementary Fig. S5). The first Q and R in the motif are not fully conserved, but the last two residues, R and Q, are fully conserved in all Hig1 type 2 members^30^.

**Fig. 3:**
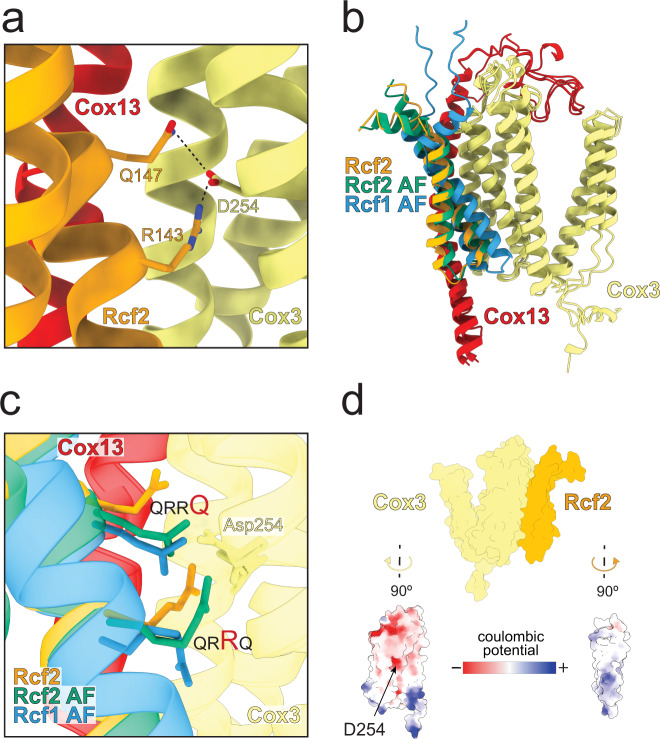
Binding of Rcf proteins. **a** A close-up view of Arg143/Gln147 of the Rcf2 QRRQ motif and Asp254 of Cox3. **b** An AlphaFold-Multimer (AF in the figure) model of Cox3, Cox13 together with either Rcf2 or Rcf1, shown together with the cryo-EM model of Rcf2 in the complex. **c** Close-up view of the structural models in panel **b**. **d** Electrostatic potential surfaces of Cox3 and Rcf2.

The *S. pombe* CIV structural model shows specific interactions of the fully conserved, second R (R143) and Q (Q147), of the QRRQ motif with Asp254 of Cox3 (Fig. 3a), which is a highly conserved residue in CIV. Previously discussed interactions with Cox13^16^ were not clearly seen in our structural model. The equivalent QRRQ motif in Rcf1 was shown to be involved in regulatory interactions with CIV^30^. To test whether Rcf1 would bind to CIV at the same position as Rcf2, we created an AlphaFold-Multimer (AF-M) model^54,55^ of Cox3, Cox13 and Rcf1. As a control, we also created an AF-M model of Cox3, Cox13 and the Hig1 domain of Rcf2. The latter model was essentially identical to the cryo-EM structural model. Comparison of the Rcf1-Cox3-Cox13 and Rcf2-Cox3-Cox13 AF-M models shows that the two HIG1 TM α-helices of both Rcf-proteins bind at the same position (Fig. 3b) and the positioning of the QRRQ motif relative to Asp254 is the same (Fig. 3c). All transmembrane regions showed plDDT >90 indicating high accuracy of the prediction. Interestingly, the conserved HIG1 fragment is found at the N-terminus of Rcf1 but at the C-terminus of Rcf2. Hence, the additional parts of the two Rcf proteins would be exposed at different positions relative to the HIG1 domain, upon association with CIV (see discussion in^33^). Thus, our data suggests that Rcf1 or Rcf2 cannot bind simultaneously to CIV and explains differences in functional effects upon binding of Rcf1 or Rcf2, respectively (reviewed in ref. ^15^).

Interactions of Rcf1 or Rcf2 with Cox3 are in part of electrostatic origin (Fig. 3d), mediated via the conserved Asp254 at Cox3 and, Arg143/Gln147 of the QRRQ motif of Rcf2 (supplementary Fig. S5). We also note that Rcf2 rather than Rcf1 is seen bound to the fully assembled and active CIV of both *S. cerevisiae*^13^ and *S. pombe*. We suggest that binding of Rcf2 at Cox3 during late assembly of CIV may involve dissociation of the Rcf1 assembly factor from the mature CIV. Binding of Rcf2 at the same position would maintain protection of the charged membrane-exposed surface of Cox3.

Hayashi et al.^56^ identified Higd1a as a regulatory component of bovine heart CIV. However, Higd1a belongs to the type 1 subgroup of the hypoxia-induced gene domains, which do not harbor a QRRQ motif. Modelling studies placed the Higd1a domain at a different position from that found for Rcf2. Furthermore, the mammalian CIV is often isolated in dimeric form^57^ and the monomer-monomer interface is found at the equivalent position of Rcf2 identified in this work.

### Ligand binding to fully reduced *S. pombe* CIV

**Fig. 4:**
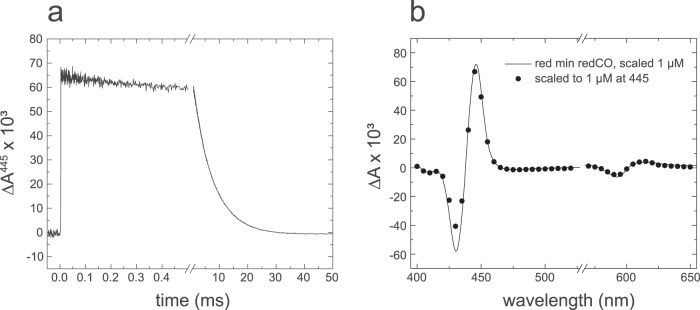
Binding of the CO ligand to reduced CIV. **a** Absorbance changes monitored at 445 nm after flash-induced CO dissociation from the fully reduced enzyme. **b** Kinetic difference spectrum (closed circles) of the CO-recombination component in (**a**) and a difference spectrum of the reduced minus reduced-CO state, recorded using a spectrophotometer. Experimental conditions: 50 mM KH_2_PO_4_, pH 8, 150 mM KCl, 0.05 % DDM, 2 mM ascorbate and 1 µM PMS (redox mediator), 1.2 mM CO. The CIV concentration was ~1 µM and all traces are scaled to 1 µM reacting enzyme. The kinetic trace in (**a**) is an average of 25 traces.

Reduced heme *a*_3_ binds CO in the absence of O_2_. The CO-Fe^2+^ bond is light sensitive and upon pulsed illumination CO dissociates (increase in absorbance at *t* = 0, Fig. 4a), followed by recombination (decrease in absorbance). The CO-recombination kinetics reflects the integrity of the catalytic site. In the *S. pombe* CIV this recombination kinetics was monophasic with a time constant of 6.9 ± 0.2 ms (average of 25 traces), which indicates that the preparation is uniform. The time constant is similar to that observed with CIVs from other species^58–60^.

Figure 4b shows a CO-recombination kinetic difference spectrum, i.e. a plot of the amplitude of the CO-recombination absorbance changes as a function of wavelength. This kinetic difference spectrum overlaps with the heme *a*_3_^2+^-minus-heme *a*_3_^2+^-CO absorbance difference spectrum, which shows that no additional processes occurred in CIV after pulsed illumination of the CIV-CO complex.

Earlier studies with the *S. cerevisiae* CIV revealed additional components in the CO-recombination kinetics, which was interpreted in terms of CIV sub-populations with different structures^60,61^. As with the mammalian CIV^58,59^, the *S. pombe* CIV displayed monophasic CO recombination (Fig. 4a) and, similar static and kinetic CO-reduced-minus-reduced difference spectra (Fig. 4b), which indicates that detergent solubilization of the *S. pombe* membrane yields a CIV preparation that is more uniform than that from *S. cerevisiae*.

### Reaction of the fully reduced *S. pombe* CIV with oxygen

**Fig. 5:**
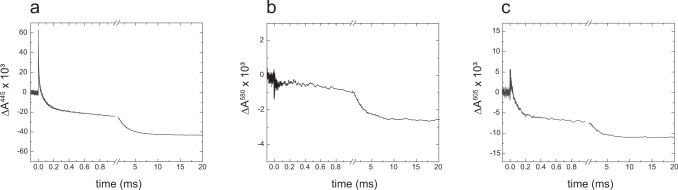
Reaction of reduced CIV with O_2_. Absorbance changes after flash photolysis of CO from the fully reduced CIV after mixing with O_2_ (at *t* = -200 ms) and flash photolysis of CO (at *t* = 0), measured at 445 nm (**a**), 580 nm (**b**) and 605 nm (**c**). Experimental conditions: 25 mM KH_2_PO_4_, pH 7.5, 150 mM KCl, 0.1 mM EDTA, 0.05 % DDM, 0.85 mM O_2_, 1.4 µM CIV. Laser artifacts due to the laser flash hitting the detector at *t* = 0 have been truncated. The data are averages over 3 (panels **a** and **c**) or 13 (panel **b**) traces. These averages were smoothed by averaging over a window of five points. All traces are scaled to 1 µM reacting enzyme.

The so-called “flow-flash” technique has been used to investigate the kinetics of the reaction of reduced CIV with O_2_^62–64^. Four-electron reduced CIV with the catalytic site blocked by CO at heme *a*_3_ is rapidly mixed with an oxygen-saturated buffer. Pulsed illumination of the CIV-CO complex results in dissociation of CO, seen as a rapid increase in absorbance at 445 nm at time zero in Fig. 5a. Dissociation of the CO ligand allows O_2_ to bind to heme *a*_3_ with a time constant of ~10 µs at 1 mM O_2_, seen as a rapid decrease in absorbance at 445 nm (Fig. 5a). Binding of O_2_ is followed in time by electron transfer from heme *a* to the catalytic site, which results in formation of the peroxy, **P**_**R**_, intermediate with a time constant of ~70 µs, seen as a further decrease in absorbance at 445 nm, 580 nm and 605 nm (Fig. 5a–c). In the next step a proton is transferred from the *n* side to the catalytic site forming the ferryl, **F**, state with a time constant of ~150 µs, seen as an increase in absorbance at 580 nm (Fig. 5b, absorbance increase in the time range 0.1–0.3 ms). This reaction is linked in time to fractional electron transfer from Cu_A_ to heme *a*, seen as a decrease in absorbance at 605 nm (Fig. 5c). In the final step the fourth electron is transferred to the catalytic site with a time constant of ~2 ms forming the oxidized state, **O** (absorbance decrease over a time range 1–5 ms). This reaction is accompanied by proton uptake from the *n* side^65^. Each of the **P**_**R**_ → **F** and **F** → **O** transition reactions is linked to proton pumping across the membrane^66,67^. The absorbance changes observed with the *S. pombe* CIV exhibit the same pattern as seen for other well-characterized CIV and the time constants of the **R** → **P**_**R**_ → **F** → **O** are in the same range as those observed with CIV from other species (Table 3). The data obtained with the *S. pombe* CIV are more similar to those of mammalian than *S. cerevisiae* CIV in that the **F** → **O** reaction is monophasic in the former two, while it is biphasic in the *S. cerevisiae* CIV^64^.

**Table 3:** Reaction of reduced CIV with O_2_.

CIV from	A → P_R_ (µs)	P_R_ → F (µs)	F → O(ms)
*S. pombe*	70	150	2.4
*S. cerevisiae*	23	90	0.4 and 6
*B. taurus* (cow)	25	70	1.0
*R. sphaeroides*	60	140	1.3

Time constants of reaction steps after oxygen binding (which was not resolved) to fully reduced *S. pombe* CIV, compared to the equivalent time constant obtained with *S. cerevisiae*^64^, bovine^63^ and *R. sphaeroides*^63^ CIV. The data with *S. cerevisiae* CIV display two components for the **F** → **O** reaction^64^.

### Detergent effects on CIV catalytic activity

The detergent GDN, used in the cryo-EM studies, harbors a steroid lipophilic group^68^, which binds and inhibits the activity of CIV^69,70^. A recent combined cryo-EM, kinetic and molecular simulations study showed that GDN and the cholesterol analogue cholesteryl hemisuccinate binds near the K pathway of the mammalian CIV and inhibits proton transfer upon reduction of CIV^71^.

**Fig. 6:**
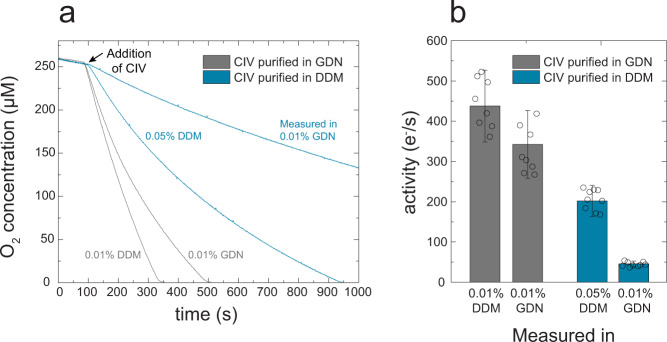
Oxygen reduction activity. **a** Concentration of O_2_ as a function of time upon addition of CIV (9 nM pre-incubated in 50 mM KH_2_PO_4_, pH 6.5, in detergent as labeled “purified in”) to the oxygraph chamber containing 10 mM ascorbate, 50 µM cyt. *c*, 0.1 mM TMPD, 50 mM KH_2_PO_4_ at pH 6.5 (with either DDM or GDN as shown specified by “measured in”). The background slope recorded before addition of CIV was subtracted from that measured after CIV addition. The differences are shown in panel **b**. Shown is the average with the standard deviations (*n* = 8-9, three independent preparations).

A similar inhibitory effect of GDN, when present in the measuring buffer, was also observed with the *S. pombe* CIV. Figure 6a shows measurements of the O_2_-reduction kinetics with samples purified in either GDN (gray) or DDM (blue), concentrated and then diluted in buffers containing different concentrations of the two detergents (referred to as “measured in” in Fig. 6a,b). For CIV that was purified in DDM the activity in DDM was 200 ± 40 e^−^ s^−^^1^ (*n* = 9, three independent preparations) (Fig. 6b, blue, 0.05 % instead of 0.01 % DDM was used in this measurement in order to keep the same detergent concentration as that used during purification and then investigate the effect of addition of GDN). However, when the DDM-purified enzyme was diluted in GDN (blue, 0.01 % GDN) the activity dropped to 40 ± 5 e^−^ s^−^^1^ (*n* = 9, three independent preparations), presumably due to binding of the steroid moiety of GDN to CIV. The GDN-purified CIV displayed a higher activity than the DDM-purified sample of 340 ± 80 e^−^ s^−^^1^ (*n* = 8, three independent preparations) in GDN (gray, 0.01 % GDN). This activity increased to 440 ± 90 e^−^ s^−^^1^ (*n* = 8, three independent preparations) upon dilution of the GDN-purified CIV in a DDM buffer (gray, 0.01 % DDM). This increase in activity is attributed to removal of bound GDN from a fraction of CIV.

The data in Fig. 6 show that GDN inhibits the CIV activity, however, the effect was more pronounced when the enzyme was initially purified in DDM than in GDN. We initially speculated that the effect could be explained if the steroid-binding site would be occupied in the native membrane and made accessible to GDN binding only after treatment with DDM. The site would remain occupied in GDN thereby preventing binding of the GDN steroid moiety. However, a detailed analysis of the cryo-EM structure of the GDN-purified CIV did not reveal any density in the steroid-binding site identified in the mammalian CIV. Hence, we instead propose that purification in DDM removes surface lipids that allow access to the steroid-binding site in the detergent-purified CIV. Purification using the weaker GDN detergent leaves the enzyme with the blocking lipids bound, which allows binding of GDN in the steroid-binding site in only a small fraction of CIV.

Upon increasing the DDM concentration from 0.01 % to 0.05 % with CIV purified in GDN, the O_2_-reduction turnover activity gradually decreased with increasing time (supplementary Fig. S6). We speculate that this behavior is due to loss of subunits Cox12 and Cox13, which are loosely bound and may dissociate upon exposure to DDM^16^. A similar behavior, referred to as suicide inactivation, was observed previously with the *R. sphaeroides* CIV, upon dissociation of subunit Cox3^72^, and with mammalian CIV^73,74^ where the behavior was attributed to loss of lipids and dissociation of Cox3.

## Conclusions

In this work, we combined the use of cryo-EM and spectroscopy to investigate the structure and function of respiratory complex IV from *S. pombe*. The data show that the reduced *S. pombe* CIV reacts with O_2_ along the same trajectory as other, previously studied CIVs. The cryo-EM structure of CIV shows that the enzyme is composed of eleven subunits. In addition, we observed a bound Hig1 domain of Rcf2, i.e. Rcf2 binds to CIV also in the absence of CIII. An AF-M model suggests that both Rcf1 and Rcf2 bind with their Hig1 domains segments at the same position at Cox3 and Cox13 of CIV. This modelling suggests that binding of Rcf1 or Rcf2 at CIV is mutually exclusive, such that Rcf2 binding requires the dissociation of the Rcf1 assembly factor. Furthermore, the results suggest that the differential functional effect of Rcf1 and Rcf2, respectively, is caused by interactions of CIV with the unique domains extending at the N-terminus for Rcf2 and the C-terminus for Rcf1.

## Methods

### Introduction of purification tags at CIV

Twin strep tags were genetically introduced at the C-termini of either Cox4, Cox5, Cox6 or Cox13 (Table S1) in the parental strain FY7507 (L972, *h*^-^, provided by the NBRP (YGRC), Japan), by a modified two-step PCR approach as in ref. ^75,76^. Flanking regions of 350–510 bp were generated in PCR1, using 0.4 µM of each primer (Table S2), 225 ng genomic DNA (isolated from FY7507 strain according to ref. ^77^, 0.2 mM dNTPs and 1.25 U pfu polymerase (Thermo Scientific). In PCR2; 50 ng of plasmid pTF277 (pFA6a-TEV-6xGly-2xStrep-KanMX, addgene plasmid 44094) was used as template, with products from PCR1 and outer flanking primers used in PCR1. A mix of Taq:pfu polymerase (15:1) was used with Taq Ammonium sulfate buffer and 2.5 mM MgCl_2_.

Transformations were performed according to ref. ^75^ with the following modifications. 0.7–0.95 µg PCR2 product was used, 40 µg single stranded DNA (Sigma, D9156) was used in each transformation and samples were incubated for 2 h at 30 °C before addition of DMSO and heat shock at 42 °C for 15 min. 200 µl of each reaction was plated on YES-4% glucose plates, which were replica plated onto YES-5% glucose plates containing 150 µg ml^−^^1^ G418 (Sigma). Six colonies of each construct were re-streaked onto fresh YES-5% glucose, G418 plates. Tag insertions were verified by colony PCR and sequencing (Eurofin genomics) using primers listed in Table S2.

### Batch growth and membrane preparation

Supplementary Fig. S7 shows growth and respiratory competence assayed using drop dilution series on YES plates containing either 2% glucose or 3% glycerol containing 0.1% glucose, which has been shown to promote *S. pombe* growth while still being dependent on respiration^78^. All tagged strains grew well on glucose, whereas the growth was impaired for Cox6^2xstrep^ under respiratory conditions (supplementary Fig. S7). Cox4^2xstrep^ and Cox5^2xstrep^ grew slightly slower on glycerol than Cox13^2xstrep^. Nevertheless, Cox5^2xstrep^ membranes contained the highest amount of tagged CIV. Therefore, Cox5^2xstrep^ strain was used for batch growth and purification of CIV.

For CIV expression, a starter culture containing 0.5% yeast extract (YE), 2% glucose and 100 µg ml^−^^1^ ampicillin was used. The pre-culture and large-scale culture contained YE-3% glycerol and 0.1% glucose. All incubation steps were carried out at 30 °C while shaking at 200 rpm. Cells were washed twice in water and stored as cell pellets at −20 °C until use.

Membranes were prepared by re-suspending cells in 0.4 M sorbitol, 50 mM KH_2_PO_4_, pH 7.4, 5 mM EDTA and 1 mM phenylmethanesulfonyl fluoride. Cell suspensions were crushed twice using a cell disrupter (Constant systems, at 38 kpsi). The cell debris was pelleted at 5500 x g for 12 min and membranes were collected at 120,000 × g, Type 45 Ti rotor (Beckman Coulter) for 1 h at 4 °C. Membrane pellets were homogenized in 50 mM KH_2_PO_4_, pH 8.0 and the protein concentration was determined using a modified Lowry assay, which contained 1% SDS in the membrane and reference samples^79^. The samples were rapidly frozen in liquid N_2_ and stored at −80 °C.

### Purification of CIV in GDN

Membranes were solubilized at 4 °C overnight at a concentration of 5 mg ml^−1^ in 25 mM KH_2_PO_4_, pH 8.0, 150 mM KCl and 1% GDN. Insoluble material was pelleted by ultra-centrifugation at 120,000 × *g*, Type 45 Ti rotor (Beckman Coulter) for 30 min at 4 °C. The supernatant was concentrated to approximately 10 ml, diluted fivefold in detergent-free buffer and concentrated again to 10 ml before applying to a StrepTactin superflow column (IBA life sciences) pre-equilibrated with 25 mM KH_2_PO_4_, pH 8 and 150 mM KCl. The column was washed with 25 mM KH_2_PO_4_, pH 8, 150 mM KCl and 0.01% GDN before elution in same buffer, supplemented with 5 mM desthiobiotin. The membrane supernatant, flow through, wash and elution fractions were all analyzed spectrophotometrically (Cary100, Agilent) to determine their protein and CIV content at 280 nm and 420 nm, respectively. Elution fractions that contained CIV were pooled and concentrated using Amicon Ultra, 100 kDa cut-off (Millipore). The sample was injected on to a Superose 6 Increase column (GE), pre-equilibrated with 25 mM KH_2_PO_4_, pH 8.0, 150 mM KCl, 0.01 % GDN at a flow rate of 0.5 ml min^–^^1^.

The concentration of CIV was determined from the reduced-minus-oxidized spectra using ε_607-630_ = 24 mM^−1^cm^−1^. Purified protein was either used immediately or frozen in liquid N_2_ and stored at −80 °C.

### Purification of CIV in DDM

Purification of CIV using the Cox5^2xstrep^ in n-dodceyl beta-maltoside (DDM, Glycon) was carried out as described above with the following modifications. Membranes were diluted to a final concentration of 2 mg ml^−1^ in 25 mM KH_2_PO_4_, pH 8.0, with a final concentration of 150 mM KCl before dropwise addition of DDM to a final concentration of 0.5% and solubilization while stirring at 4 °C for 45 min, before removing insolubilized material. All purification buffers contained 0.05% DDM instead of GDN.

### Grid preparation and Cryo-EM

GDN-purified CIV (3 µl) at a concentration of ~4 mg ml^−1^ was applied to holey carbon film coated copper EM grids (C flat 2/2 3 C T50) that had been glow-discharged in air (120 s, 20 mA using PELCO easiGlow). Grids were blotted for 3 s at 4 °C and 100 % humidity before rapid freezing in liquid ethane with a Vitrobot Mark IV (Thermo Fisher Scientific). Cryo-EM data were collected using a Titan Krios G3 electron microscope (Thermo Fisher Scientific) operated at 300 kV, equipped with a Gatan BioQuantum energy filter and a K3 Summit direct electron detector (AMETEK). Automated data collection was done with the EPU software package (Thermo Fisher Scientific). A dataset of 9289 movies, each consisting of 40 exposure fractions was collected at a nominal magnification of 105,000x, corresponding to a calibrated pixel size of 0.86 Å. The camera exposure rate and the total exposure of the specimen were 13.0 e^−^ pixel^−1^ s^−1^ and ~49 e^−^ Å^−2^, respectively (Table 2).

### Cryo-EM Image Processing

All image analysis was performed with cryoSPARC v2^80^. Movies were aligned in patches and contrast transfer function (CTF) parameters were estimated in patches with a 7 × 7 grid. The dataset was manually curated to remove movies with devitrification, large cracks in the ice, or poor CTF fit parameters, reducing the dataset size to 8144 movies. Templates for particle selection were generated by 2D classification of manually selected particle images. A total of 2,609,959 particles were selected, images were corrected for local motion^81^ and extracted in 256 × 256 pixel boxes. The dataset was first cleaned with 2D classification and then with several rounds of ab initio 3D classification and heterogeneous refinement, taking only the classes that corresponded to CIV after each round. This procedure further reduced the size of the dataset to 127,659 particle images, which were subject to Non-uniform refinement to produce the final map.

### Model building

Starting models were generated from the AlphaFold Protein Structure Database^82^. Sequence and AlphaFold starting model for Cox1 was Uniprot entry P07657 from strain L972 with 537 residues. Density for an additional Tyr at position 400 was found in the map. This residue is present in Cox1 sequences for Uniprot entry A0A516IL59, A0A516ILY3, A0A516IKQ3, consisting of 538 residues. The additional Tyr400 was included in the model. Refinement of the model was performed in Coot^83^. Final refinement and calculation of atomic displacement parameters were done with real space refine in Phenix^84^.

### Gel electrophoresis

NativePAGE 4–16% (Invitrogen) was used for Blue Native PAGE (BN), performed according to the manufacturer´s instruction using Native PAGE 1X running buffer at the anode and NativePAGE 1X running buffer supplemented with NativePAGE 1X cathode buffer additive as cathode buffer (Invitrogen). The gel was run at 4 °C for 60 min at 150 V, before exchanging the cathode buffer to the anode buffer and running for an additional 40 min at 250 V. The gel was then stained with Coomassie Brilliant Blue. The bands corresponding to CIV from BN PAGE were subjected to mass spectrometry (LC-MS/MS), using digestion with trypsin and a 35 min gradient.

### Oxygen reduction activity

Steady state turnover activity was measured using a Clark type oxygen electrode (Hansatech) in 50 mM KH_2_PO_4_, pH 6.5, supplemented with DDM or GDN (see figure legends). Equine cytochrome *c* (Sigma)(50 µM), 10 mM ascorbate and 0.1 mM TMPD were mixed in before addition of CIV at a concentration of 9 nM. Background oxygen consumption was recorded before addition of CIV and subtracted from the slope measured after addition of CIV.

### CO-recombination kinetics

CIV was added at a final concentration of 1 µM to a buffer composed of 50 mM KH_2_PO_4_ (pH 8), 150 mM KCl, 0.05% DDM and 1 µM PMS (redox mediator) in a Thunberg cuvette. The atmosphere in the cuvette was exchanged for nitrogen on a vacuum line, before addition of 2 mM ascorbate. Reduction was monitored spectrophotometrically (Cary 4000) before exchange of the N_2_ atmosphere for CO. The CO recombination kinetics was monitored using a flash photolysis set up (Applied Photophysics) by monitoring absorbance changes upon photo dissociation of the CO ligand using a 10-ns laser flash (Quantel Brilliant B, Nd-YAG, 532 nm). Traces were scaled to 1 µM reacting enzyme using ε_445_ 67 mM^−1^cm^−1^^85^.

### Reaction of fully reduced CIV with oxygen

The CIV sample was prepared as for measurements of the CO-recombination kinetics, with the following exceptions. The enzyme was diluted to 5 µM in 25 mM KH_2_PO_4_, pH 7.5, 150 mM KCl, 0.1 mM EDTA and 0.05 % DDM and reduced using 2 mM ascorbate and 1 µM PMS. The fully reduced protein was mixed at a 1:2.5 ratio with an O_2_-saturated buffer (25 mM KH_2_PO_4_, pH 7.5, 150 mM KCl, 0.1 mM EDTA, 0.05% DDM) in a stopped-flow apparatus (Applied Photophysics). At 200 ms after mixing, the CO ligand was dissociated by a 10-ns laser flash (Quantel Brilliant B, Nd-YAG, 532 nm). Absorbance changes were monitored as above at individual wavelengths (see figure legend to Fig. 5). Traces were averaged, smoothed (see Figure legend) and fitted with a sum of exponential functions.

### Reporting summary

Further information on research design is available in the Nature Portfolio Reporting Summary linked to this article.

## Supplementary information

Supplementary Information

Description of Additional Supplementary Files

Supplementary Data 1

Reporting Summary

## Data Availability

The cryo-EM map and atomic coordinates have been deposited in the Electron Microscopy Data Bank and Protein Data Bank, under accession numbers EMD-16491 and PDB 8C8Q (also available as Supplementary Data 1), respectively.
