# Supplementary material for: Cryo-EM structure and function of S. pombe complex IV with bound respiratory supercomplex factor
Source: Commun Chem. 2023 Feb 16;6:32. doi: 10.1038/s42004-023-00827-3 (PMC9935853; doi:10.1038/s42004-023-00827-3)
Supplement: Supplementary file 1 — Supplementary Information [file 42004_2023_827_MOESM1_ESM.pdf]

Supplementary information

Cryo-EM structure and function of *S. pombe* respiratory complex IV with bound respiratory supercomplex factor

Agnes Moe[1], Pia Ädelroth[1], Peter Brzezinski[1*] and Linda Näsvik Öjemyr[1,2*]

[1]Department of Biochemistry and Biophysics, The Arrhenius Laboratories for Natural Sciences, Stockholm University, SE-106 91 Stockholm, Sweden.

[2]Present address: Xbrane Biopharma AB, Retzius väg 8, SE-171 65 Solna, Sweden.

[*]Correspondence: Linda Näsvik Öjemyr, linda.nasvik-ojemyr@xbrane.com or Peter Brzezinski, peterb@dbb.su.se

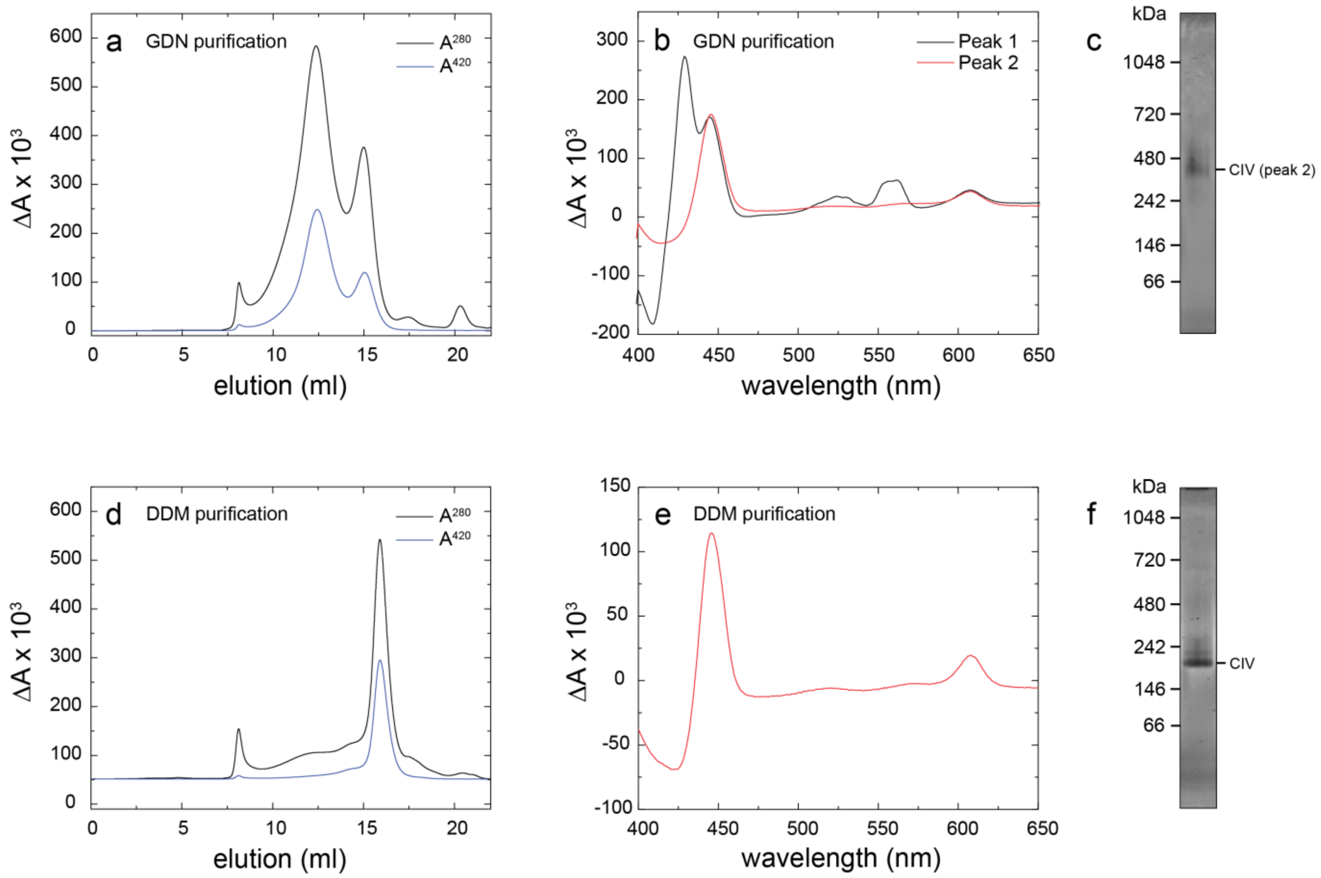

Supplementary Figure S1. Purification of *S. pombe* CIV. (**a**) Elution profile from size-exclusion chromatography with a Superose 6 Increase 10/300 GL column in 150 mM KCl, 25 mM KPi buffer, pH 8.0, 0.01 % GDN. Elution was followed at 280 nm (total protein) and 420 nm (hemes). Hemes were found in two of the peaks. (**b**) Dithionite reduced-minus-oxidized difference spectra of the two peaks from size exclusion chromatography (see panel A) showing the presence of supercomplexes (SC), eluted in the first peak, and free CIV in the second peak. (**c**) BN-PAGE of the GDN-purified CIV (peak 2 in panel A). The band corresponding to CIV was subjected to mass spectrometry where Rcf2 was detected with a score of ~18, sequence coverage of ~27 % and 9 peptides were identified. Overall, the data suggests that the processed, C-terminal form of Rcf2 is part of the mature CIV[1,2]. Even though peptides originating from the N-terminal domain were detected in the mass spectrometry analysis, it is unlikely that these peptides originate from an intact Rcf2; these fragments may rather be unspecifically attached to CIV. (**d**) Elution profile from size-exclusion chromatography in 150 mM KCl, 25 mM KPi buffer, pH 8.0, 0.01 % DDM. (**e**) Dithionite reduced-minus-oxidized difference spectra of the peak from size exclusion chromatography (see panel D) showing the presence of free CIV. (**f**) BN-PAGE of the DDM-purified CIV.

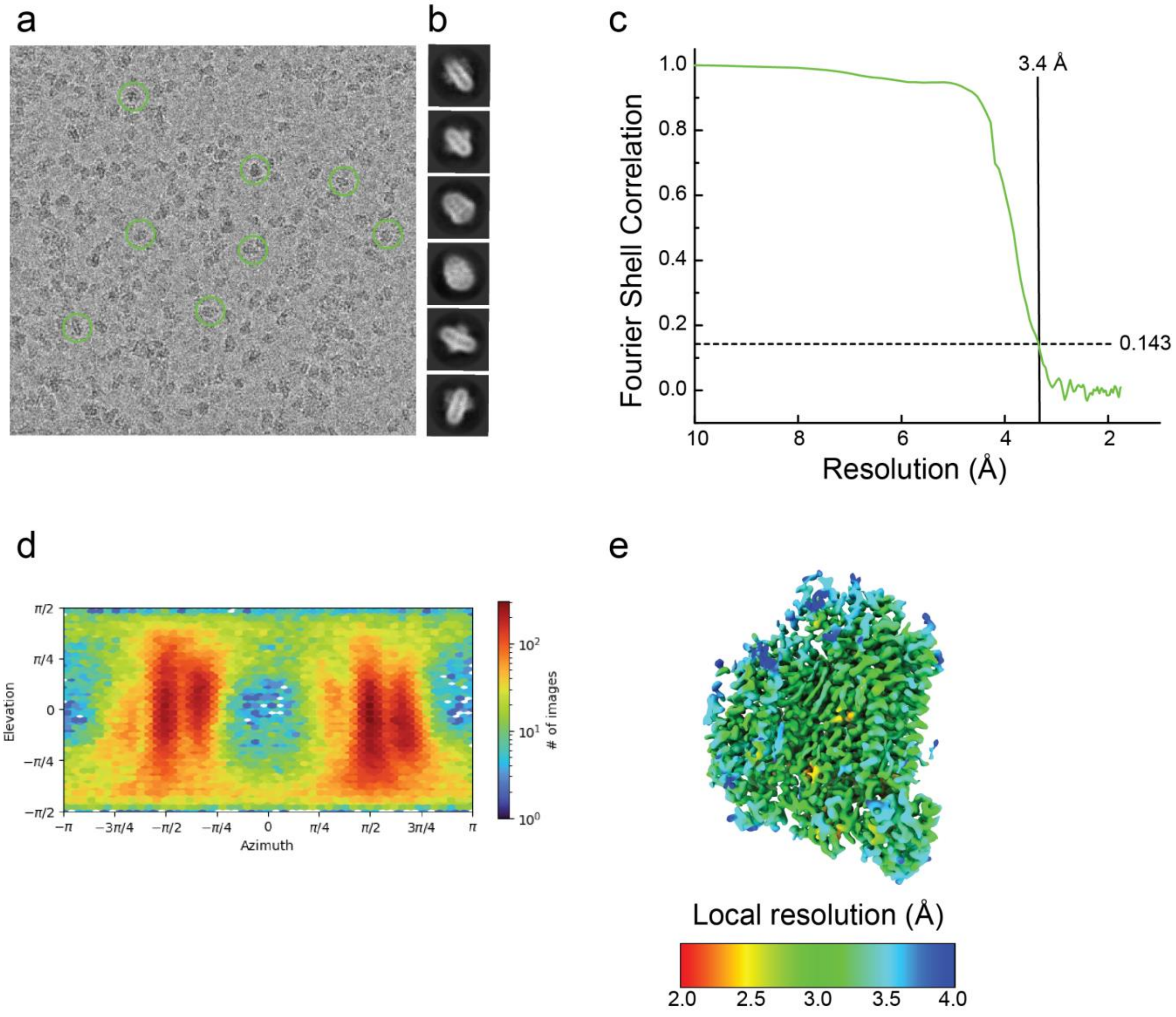

Supplementary Figure S2. Cryo-EM validation. (**a**) Example micrograph. (**b**) 2D class averages. (**c**) Fourier shell correlation (FSC) curve after refinement, corrected for the effects of masking. (**d**) Viewing direction distribution for particle images. (**e**) Local resolution map.

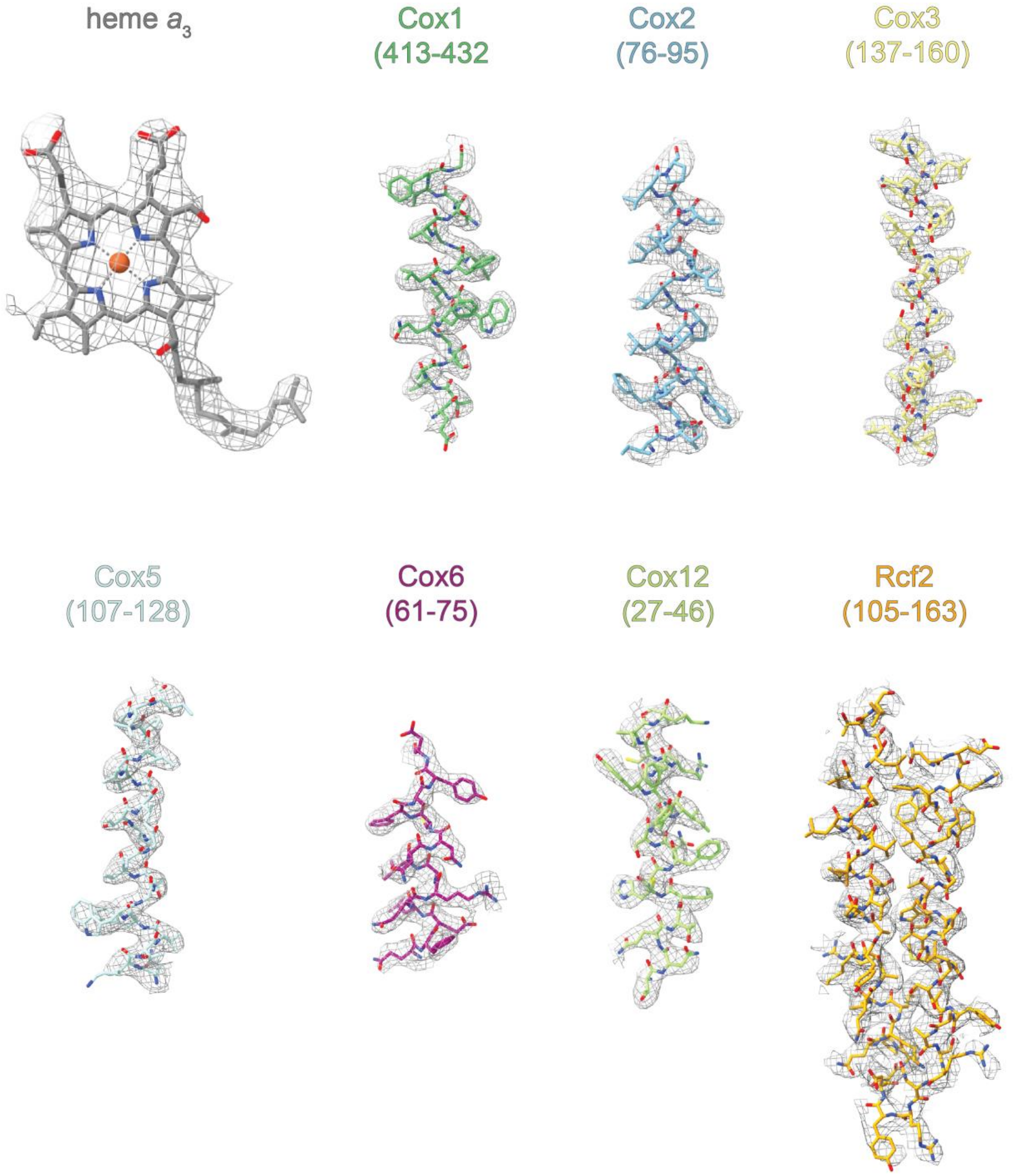

Supplementary Figure S3. Model in map fit. Representative atomic model and experimental map for selected regions. Both transmembrane helices from Rcf2 are shown.

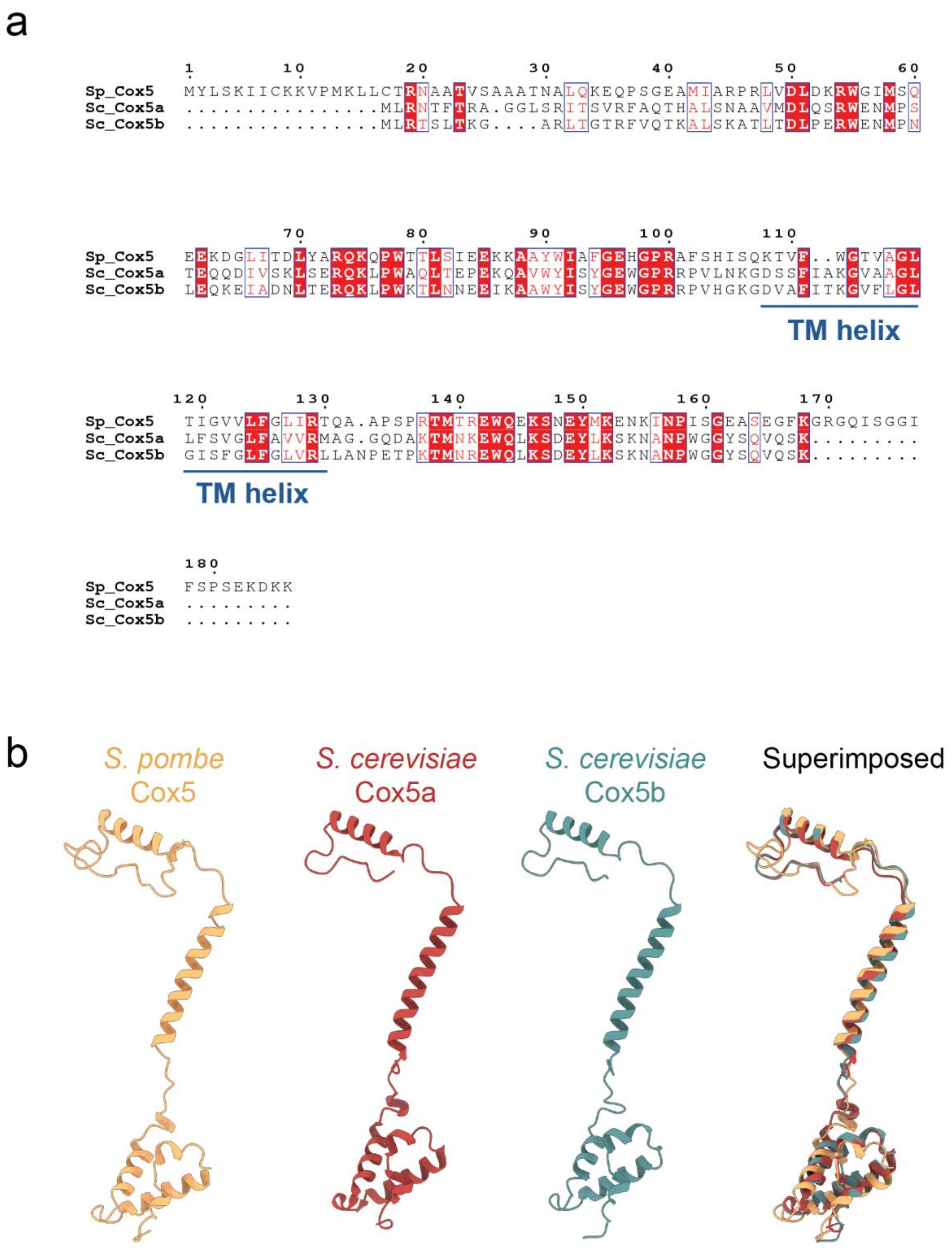

Supplementary Figure S4. Comparison of Cox5 from different species. (**a**) Sequence alignment of Cox5 from *S. pombe* (Uniprot: O74988), and Cox5a (P00424) and Cox5b (P00425) from *S. cerevisiae*, performed using ClustalO [3]. Cox5 from *S. pombe* has 53% and 58% sequence similarity with Cox5a and Cox5b, respectively. The numbers above the sequences correspond to the Cox5 sequence from *S. pombe*. The position of the transmembrane helix is indicated in blue. The image was generated in ESPript [4]. (**b**) Models of Cox5 from *S. pombe*, Cox5a (from PDB 6HU9) and Cox5b (from PDB 6T15) from *S. cerevisiae*, as well as all three models superimposed.

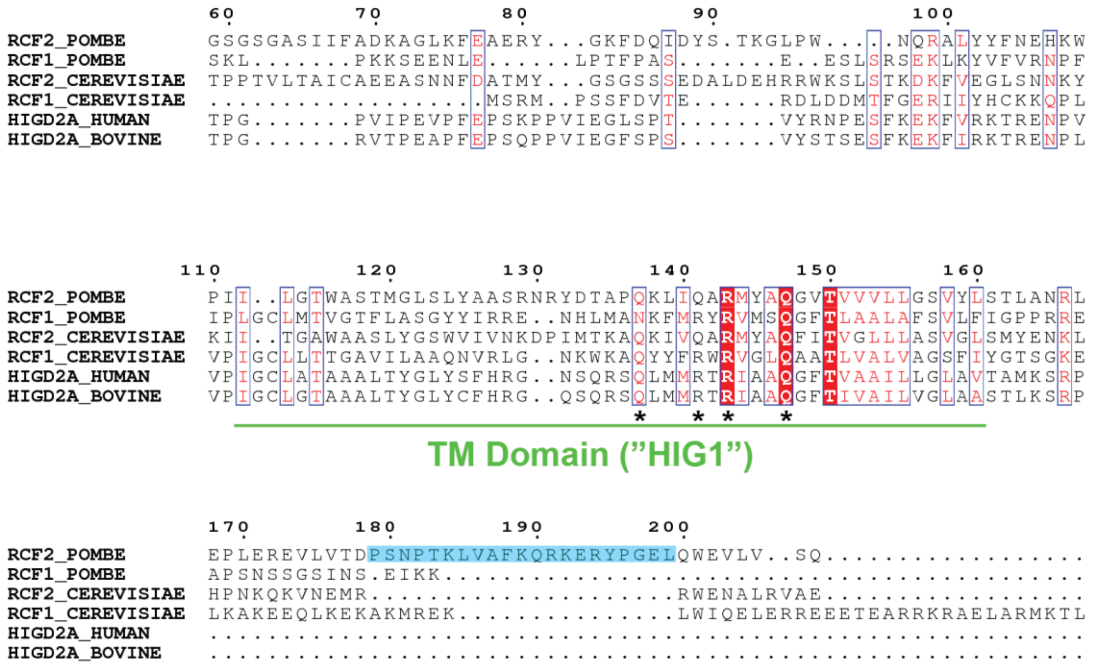

Supplementary Figure S5. Sequence alignment of Rcf2. Sequence alignment of Rcf2 (Uniprot: Q9P3B2) and Rcf1 (Q9UTB1) from *S. pombe*, Rcf2 (P53721) and Rcf1 (Q03713) from *S. cerevisiae*, as well as human (Q9BW72) and bovine (Q05AT5) Higd2a, performed using ClustalO [3]. The numbers above the sequences correspond to the Rcf2 sequence from *S. pombe*. The alignment shows only a fraction of Rcf2 and Rcf1, highlighting the HIG1 domain in green. The conserved QRRQ motif is indicated with stars underneath the sequences. The additional loop in *S. pombe* Rcf2 consisting of 21 residues, not present in *S. cerevisiae*, is indicated in blue. The image was generated in ESPript [4].

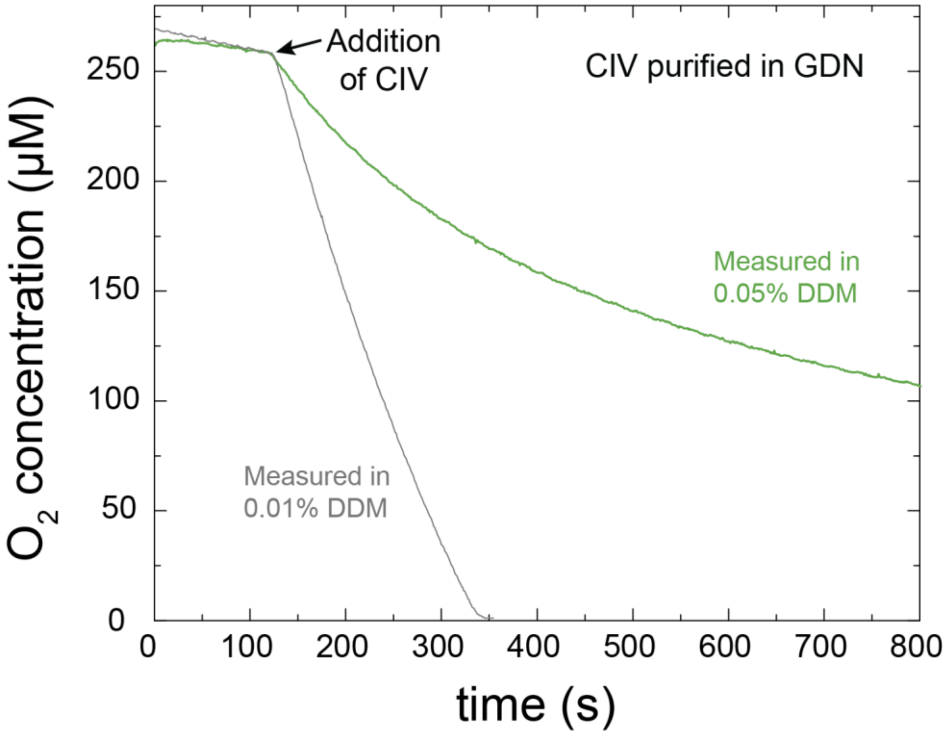

Supplementary Figure S6. Oxygen reduction activity. Concentration of O_2 as a function of time upon addition of CIV (9 nM pre-incubated in 50 mM KH_2PO_4, pH 6.5, 0.01 % GDN) to the oxygraph chamber containing 10 mM ascorbate, 50 µM cyt. *c*, 0.1 mM TMPD 50 mM KH_2PO_4 at pH 6.5 with either 0.01 % or 0.05 % DDM. The background slope recorded before addition of CIV was subtracted from that measured after CIV addition.

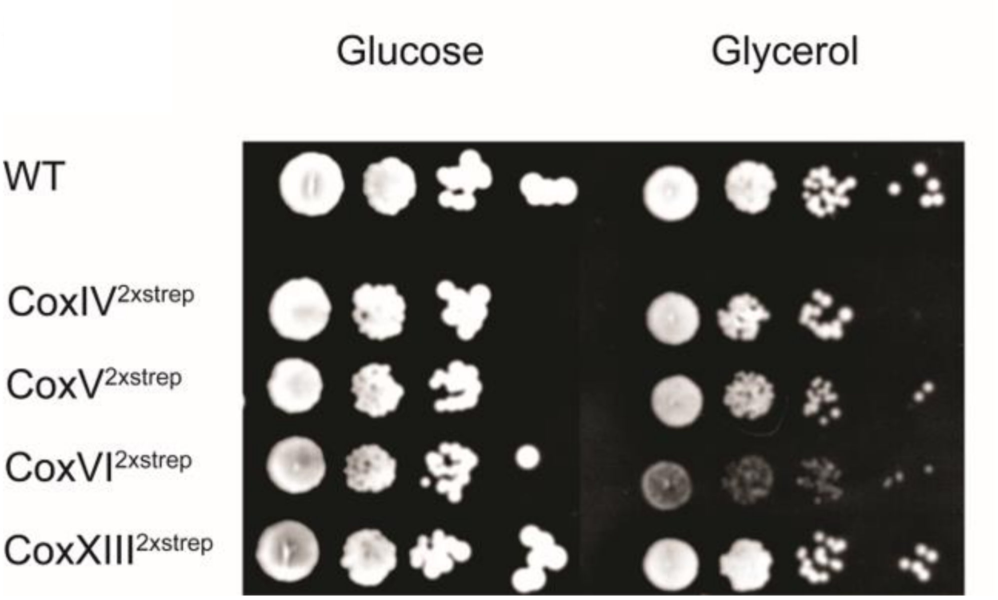

Supplementary Figure S7. Growth and respiratory competence assayed using drop dilution. A 10-fold serial dilution growth assay on fermentable and non-fermentable medium, YES agar plates (0.5 % YE, pH 5.4, SP supplements (Formedium, PSU0101) containing 2 % glucose or 3 % glycerol and 0.1 % glucose). Plates were incubated at 30 °C

.

Table S1. Strains used in this work

Strain name	Phenotype	Reference
FY7507 (L972)	h-,	NBRP (YGRC), Japan
LNO11	h-, cox4-TEV2xstrep-kanMX	This work
LNO12	h-, cox5- TEV2xstrep-kanMX	This work
LNO13	h-, cox13-TEV2xstrep -kanMX	This work
LNO14	h-, cox6- TEV2xstrep -kanMX	This work

Table S2. Primers used in this work

Primers for PCR 1		
Name	Sequence*	Gene
SpK-001	GGATGCTTCTCGTAAAGGC	Cox4-5' fw
SpK-003	CCAGTTTAAACGAGCTCGAATTCTATGCTTACCAATCTTCCATATCC	Cox4-3' fw
SpK-004	GATCATATTTCCGCATGACTAC	Cox4-3'-rev
Spk-005	GATCGGTGTCGTATTGTTTG	Cox5-5' fw
SpK-007	CCAGTTTAAACGAGCTCGAATTCGAAATTCCCACATTTTGAGTTC	Cox5-3' fw
SpK-008	GACTTATTCACGATAAGCTGA	Cox5-3'-rev
SpK-009	CTCCAAAGAGGCCTAAATA	Cox6-5' fw
SpK-011	CCAGTTTAAACGAGCTCGAATTCGTCCGATTAAGTTTCTTGATC	Cox6-3' fw
SpK-012	GTATGAGAAATGGTATTGGCA	Cox6-3'-rev
SpK-013	CCTGGTTATAGGTATGAAGAA	Cox13-5' fw
SpK-015	CCAGTTTAAACGAGCTCGAATTCGCATAGCGTTCTTTAAGCC	Cox13-3' fw
SpK-016	CACTGTACGTCGCTAAACA	Cox13-3'-rev
SpK-029	TTAATTAACCCGGGGATCCGATGACTGTGTTCAGCGTTGG	Cox4-5' rev
SpK-030	TTAATTAACCCGGGGATCCGTTTCTTGTCCTTTTCGGAGG	Cox5-5' rev
Spk-031	TTAATTAACCCGGGGATCCGCTTAAAAAGATCCTCCTTCAA	Cox6-5' rev
SpK-032	TTAATTAACCCGGGGATCCGTTCATCATCCTTCTTTAAATGATTG	Cox13-5' rev
*) underlined sequence for PCR2, complementary sequences to pTF277 (pFA6a-TEV-6xGly-2xStrep-KanMX)		
Primers for colony PCR		
SpC-001	CGGATGTGATGTGAGAACTGTATCCTAGC	kan cassette rev
SpC-017	GCTGCCGATACATTTTAGGGC	Cox4-fw
SpC-020	GAGTGCTGCTGCTACCAAC	Cox5-fw
SpC-022	GGAATGCTGGGAACTGCAAAG	Cox6-fw
SpC-023	GCAGTGGTATGTGAATTGTGGG	Cox13-fw
Primers for sequencing		
SpK-024	GCTCAAGAATTAGCCAACGC	Cox4
SpK-026	CATGATCGCTCGTCCTC	Cox5
SpK-022	GAGTACCCAATACTGAAAG	Cox6
SpK-013	CCTGGTTATAGGTATGAAGAA	Cox13

Supplementary References

1 Hartley, A. M., Meunier, B., Pinotsis, N. & Maréchal, A. Rcf2 revealed in cryo-EM structures of hypoxic isoforms of mature mitochondrial III-IV supercomplexes. *Proc. Natl. Acad. Sci. USA* **117**, 9329-9337, doi:10.1073/pnas.1920612117 (2020).

2 Römpler, K. *et al.* Overlapping role of respiratory supercomplex factor Rcf2 and its N-terminal homolog Rcf3 in *Saccharomyces cerevisiae*. *J. Biol. Chem.* **291**, 23769-23778, doi:10.1074/jbc.M116.734665 (2016).

3 Sievers, F. *et al.* Fast, scalable generation of high-quality protein multiple sequence alignments using Clustal Omega. *Molecular Systems Biology* **7**, 539, doi:https://doi.org/10.1038/msb.2011.75 (2011).

4 Robert, X. & Gouet, P. Deciphering key features in protein structures with the new ENDscript server. *Nucleic Acids Research* **42**, W320-W324, doi:10.1093/nar/gku316 (2014).
