# Supplementary material for: Cryo-EM structure and function of S. pombe complex IV with bound respiratory supercomplex factor
Source: Commun Chem. 2023 Feb 16;6:32. doi: 10.1038/s42004-023-00827-3 (PMC9935853; doi:10.1038/s42004-023-00827-3)
Supplement: Supplementary file 2 — Description of Additional Supplementary Files [file 42004_2023_827_MOESM2_ESM.pdf]

Description of Additional Supplementary Files

File name: Supplementary Data 1

Description: Structural model deposited to the Protein Data Bank (PDB ID: 8C8Q)
